# Cytoreductive Surgery and Hyperthermic Intraperitoneal Chemotherapy for Colorectal Peritoneal Metastases: A Summary of Key Clinical Trials

**DOI:** 10.3390/jcm11123406

**Published:** 2022-06-14

**Authors:** Kevin M. Turner, Mackenzie C. Morris, Davendra Sohal, Jeffrey J. Sussman, Gregory C. Wilson, Syed A. Ahmad, Sameer H. Patel

**Affiliations:** 1 Department of Surgery, University of Cincinnati College of Medicine, Cincinnati, OH 45267-0558, USA; turne2kn@ucmail.uc.edu (K.M.T.); morri2m8@ucmail.uc.edu (M.C.M.); 2 Department of Internal Medicine, Division of Hematology & Oncology, University of Cincinnati College of Medicine, Cincinnati, OH 45267-0558, USA; sohalda@ucmail.uc.edu; 3 Department of Surgery, Division of Surgical Oncology, University of Cincinnati College of Medicine, 231 Albert Sabin Way (ML 0558), Cincinnati, OH 45267-0558, USA; sussmaj@ucmail.uc.edu (J.J.S.); wilsong3@ucmail.uc.edu (G.C.W.); ahmadsy@ucmail.uc.edu (S.A.A.)

**Keywords:** colorectal peritoneal carcinomatosis, cytoreductive surgery, hyperthermic intraperitoneal chemotherapy (HIPEC)

## Abstract

The peritoneal cavity is a common site of metastatic spread from colorectal cancer (CRC). Patients with peritoneal metastases (PM) often have aggressive underlying tumor biology and poor survival. While only a minority of patients with CRC have potentially resectable disease, the high overall incidence of CRC makes management of PM a common clinical problem. In this population, cytoreductive surgery (CRS)-hyperthermic intraperitoneal chemotherapy (HIPEC) is the only effective therapy for appropriately selected patients. In this narrative review, we summarize the existing literature on CRS-HIPEC in colorectal PM. Recent prospective clinical trials have shown conflicting evidence regarding the benefit of HIPEC perfusion in addition to CRS. Current strategies to prevent PM in those at high-risk have been shown to be ineffective. Herein we will provide a framework for clinicians to understand and apply these data to treat this complex disease presentation.

## 1. Introduction

Colorectal cancer (CRC) is the fourth most common cancer in the United States, estimated to be responsible for 52,980 deaths in 2021 [1]. Following hematogenous metastases to the liver, peritoneal metastases (PM) is the second most common site in CRC, thought to be secondary to transcoelomic spread due to tumor perforation, full thickness invasion of the bowel wall, or iatrogenically during resection [2,3]. It is estimated that PM occurs in approximately 7–9% of all patients with CRC, with roughly half presenting synchronously with the primary tumor, and the other half developing metachronous peritoneal disease [4,5]. Of patients with CRC PM, between one third and one half have disease isolated to the peritoneal cavity that is potentially amenable to treatment with a curative approach [5,6]. Even with such small percentages, the high incidence of CRC equates to roughly 2000 potentially treatable cases of PM that will occur in the US this year alone.

Cytoreductive surgery (CRS)-hyperthermic intraperitoneal chemotherapy (HIPEC), first described in 1980 and popularized by Dr. Sugarbaker and colleagues, is the only effective therapy for CRC PM [7,8]. The peritoneum can be thought of as an organ, and therefore, can be resected during the CRS portion of the operation, with residual disease quantified by the completeness of cytoreduction (CCR) score [9]. Heated intraperitoneal chemotherapy is then instilled into the peritoneal cavity and allowed to circulate for 30–120 min. Hyperthermia has been shown to both directly kill tumor cells and enhance the efficacy of the chemotherapeutic agent by increasing the depth of penetration [9,10]. There are a wide variety of potential intraperitoneal chemotherapeutics, with mitomycin C (MMC) the most commonly used agent in the United States, whereas oxaliplatin is more commonly used in Europe [11]. Together, CRS-HIPEC act synergistically to optimize tumor destruction and potentially improve survival among patients with peritoneal malignancies. In this review, we will analyze recent clinical trials for patients with PM secondary to colorectal cancer.

## 2. Clinical Trials Evaluating the Efficacy of CRS-HIPEC for Colorectal Cancer

### 2.1. Netherlands Trial

**Figure 1:**
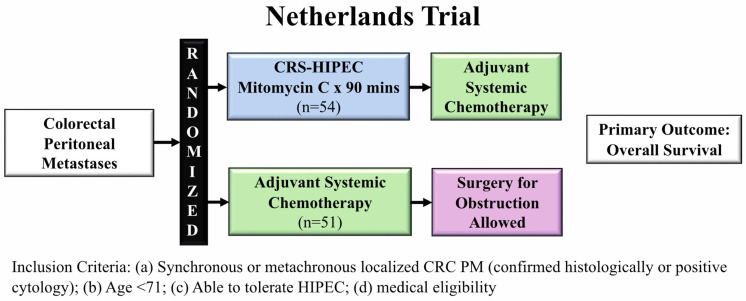
Netherlands trial schema comparing CRS-HIPEC to adjuvant systemic therapy evaluating overall survival.

Verwaal et al. conducted a randomized control trial (RCT) in the Netherlands examining whether aggressive cytoreduction with HIPEC was superior to standard systemic therapy in patients with PM secondary to CRC. Between 1998 to 2001, 105 patients with histologically proven PM from CRC (synchronous + metachronous) were randomized at diagnosis for either standard of care systemic chemotherapy or CRS-HIPEC followed by systemic chemotherapy using the same regimen (Figure 1) [12].

Systemic chemotherapy included weekly 5-fluorouracil (5-FU) and leucovorin for 26 weeks. CRS was performed with the goal to remove all macroscopic disease, although patients were still enrolled even if they had incomplete cytoreduction. The HIPEC regimen utilized in this trial was MMC for 90 min. Initial study results demonstrated improvement in overall survival (OS) by 10 months in the CRS-HIPEC arm (median OS: 22.3 months vs. 12.6 months, *p* = 0.032). After a median follow up of almost 8 years, updated results showed both improved progression-free and disease-specific survival in the CRS-HIPEC arm [13]. In the cohort who underwent complete cytoreduction, 5-year OS was 45%, demonstrating a high “cure rate” for metastatic disease.

This trial was important because it was the first RCT to examine CRS-HIPEC with adjuvant systemic therapy compared to standard of care chemotherapy. In spite of the impressive findings, the study was met with criticism. As an example, despite the study’s intent of enrolling only patients with CRC, 17% (*n* = 18) of patients actually had appendiceal cancer. The systemic regimen utilized in the study was single agent 5-FU, which was no longer the standard of care by the time the study had completed accrual, having been replaced by multi-agent regimens (FOLFOX and FOLFIRI). Although these regimens could have increased survival, potentially negating the benefit of CRS-HIPEC, proponents of the study argued that the survival benefit from the newer regimens would have affected both arms of the study, preserving the benefit of CRS-HIPEC. Finally, the treatment related mortality in the CRS-HIPEC arm was 8%, which was substantially higher than what is typically seen in practice. One potential reason was the selection of patients with extensive peritoneal disease due to difficulties in staging the peritoneum, a cohort of patients that has uniformly poor short- and long-term outcomes.

However, despite these criticisms, this study supported the use of CRS-HIPEC in patients with CRC PM.

### 2.2. PRODIGE 7

**Figure 2:**
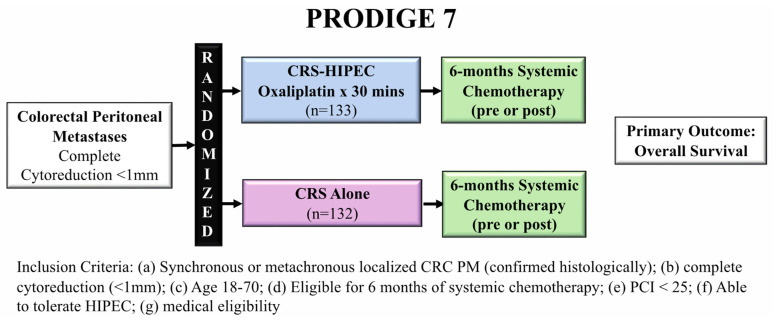
PRODIGE 7 trial schem comparing CRS-HIPEC to CRS alone evaluating overall survival.

PRODIGE 7 was a French phase III clinical trial that randomized 265 patients with CRC PM who had a peritoneal carcinomatosis index (PCI) < 25 and underwent complete cytoreduction (<1 mm residual tumor) into two arms: CRS-HIPEC vs. CRS alone (Figure 2) [14].

The HIPEC arm used bidirectional therapy with intravenous 5-FU and folinic acid 20 min prior to infusion of intraperitoneal oxaliplatin for 30 min. Importantly, all patients were required to be eligible for 6 months of systemic chemotherapy (pre-, post-, or peri-operatively), with both groups receiving a median of 6 cycles of chemotherapy and more than 40% of patients were treated with pre-operative oxaliplatin. Overall survival, the primary endpoint of the trial, was similar between the CRS-HIPEC and CRS alone arms at the final follow-up (41.7 months vs. 41.2 months, respectively, *p* = 0.99). Although mortality and 30-day morbidity were similar between the two arms, 60-day grade-3 or worse adverse events were more frequent in the CRS-HIPEC arm (26% vs. 15%, *p* = 0.035). These late complications were inherently different from the early surgical complications, being more medical in nature and were felt to be due to the HIPEC perfusion protocol. The authors concluded that due to the similar survival outcomes and increased morbidity, CRS alone, without HIPEC, should be the standard of care for patients with CRC PM treated with curative intent. PRODIGE 7 did not support the use of HIPEC with high-dose oxaliplatin over 30 min, potentially changing the paradigm for how we approach these patients, and there are many relevant criticisms of this trial.

The OS seen in this study, 41 months, was significantly longer than expected based on previous results (22.3 months in Verwaal et al. [12]), likely due to a multitude of factors, including the requirement for complete cytoreduction, a heavily pre-treated cohort, and improved modern combination chemotherapy regimens [14]. This long OS may have masked any survival benefit HIPEC could have provided. Additionally, the power analysis was dependent on an expected 18-month difference in survival with HIPEC, likely overestimating this presumed benefit. Twelve percent of patient in the CRS arm crossed over to CRS-HIPEC, compared to 7% in the CRS-HIPEC arm, confounding the interpretation of overall survival, the primary endpoint in this trial [15]. Additionally, the pathologic response to neoadjuvant chemotherapy was not taken into account, which may represent a selection criteria to identify patients who may derive the most benefit from HIPEC therapy [16]. In a posthoc subset analysis, CRS-HIPEC seemed to show a survival benefit in patients with a PCI between 11–15, identifying a potential subgroup with a poor response to systemic therapy or initially extensive disease that may benefit the most from HIPEC. The reliance on a systemic chemotherapy backbone for treating CRC PM when more than 30% of patients discontinued systemic therapy underscores that this strategy may not be optimal for all patients [16].

The HIPEC regimen utilized in PRODIGE 7 has also been questioned for the choice of intraperitoneal agent, dosing of the intravenous chemotherapy, and duration of the perfusate. The choice of oxaliplatin instead of MMC used by Verwaal et al. was supported by retrospective evidence that showed similar outcomes between these two perfusates [17]. However, pre-clinical evidence has also suggested that induced oxaliplatin chemoresistance from the high rate of oxaliplatin pre-treatment (more than 40% of all patients) could have made the HIPEC regimen ineffective, maintaining a benefit of other perfusion agents, namely MMC [18,19,20]. Oxaliplatin monotherapy is associated with response rates in colorectal cancer as low as 20%; however, combining high-dose 5-FU and folinic acid doubles this rate [21,22]. The dose of 5-FU used in the PRODIGE 7 trial was potentially inadequate to induce this synergism, limiting the efficacy of this regimen [15]. The short-duration of intraperitoneal oxaliplatin may have impacted the efficacy of the HIPEC regimen by reducing the physical elimination of tumor cells, limiting the chemotherapeutic exposure time, and leading to inadequate duration of hyperthermia for both primary cancer control and augmentation of the cytotoxic chemotherapeutic [15,23].

Although there are clear limitations of this study requiring future clinical trials to resolve, this trial provides support for CRS alone in patients with CRC PM compared to CRS in combination with the bidirectional HIPEC regimen tested (30 min of intraperitoneal oxaliplatin and intravenous 5-FU + folinic acid).

### 2.3. Conclusion to Efficacy of CRS-HIPEC

The Netherlands trial and PRODIGE 7 support the benefit of cytoreductive surgery in CRC PM. Although PRODIGE 7 demonstrated that perfusion with high dose, short duration oxaliplatin is not recommended, future clinical trials are needed to evaluate the effectiveness of HIPEC perfusion and the ideal regimen for therapeutic effect.

## 3. Clinical Trials Evaluating Early Interventions in Patients at High-Risk for Colorectal Peritoneal Metastases

### 3.1. COLOPEC

**Figure 3:**
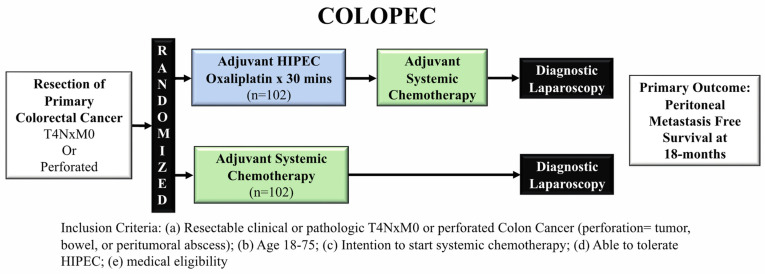
COLOPEC trial schema comparing adjuvant HIPEC to adjuvant systemic therapy in high-risk patients for peritoneal metastases.

COLOPEC was a Phase III RCT evaluating adjuvant, prophylactic HIPEC in patients with locally advanced colon cancer defined as having T4NxM0 or perforated disease without PM (Figure 3) [24].

COLOPEC randomized 204 patients to adjuvant HIPEC with systemic chemotherapy or systemic chemotherapy alone. The primary endpoint was 18-month peritoneal metastases-free survival. A diagnostic laparoscopy was performed on patients who showed no signs of peritoneal recurrence at 18 months. Adjuvant HIPEC consisted of systemic intravenous 5-FU and leucovorin given with intraperitoneal infusion of oxaliplatin for 30 min (bidirectional therapy), either during the time of primary tumor resection or within 5–8 weeks post-operatively. Over the course of the study, 19% of patients developed PM: 8% during follow up, 9% during surgical exploration, and 2% during laparoscopy performed at 18 months. Peritoneal metastases-free survival was similar between the arms at 18 months (80.9% HIPEC arm vs. 76.2% systemic chemotherapy, logrank one-sided *p* = 0.28). Importantly, nine patients found to have PM early after resection of the primary tumor likely had synchronous peritoneal metastases that were not identified during the index operation, potentially obscuring the benefit of an adjuvant HIPEC regimen.

The authors concluded that in patients with locally advanced or perforated CRC, adjuvant HIPEC with oxaliplatin did not improve 18-month peritoneal metastases-free survival.

### 3.2. PROPHYLOCHIP-PRODIGE 15

**Figure 4:**
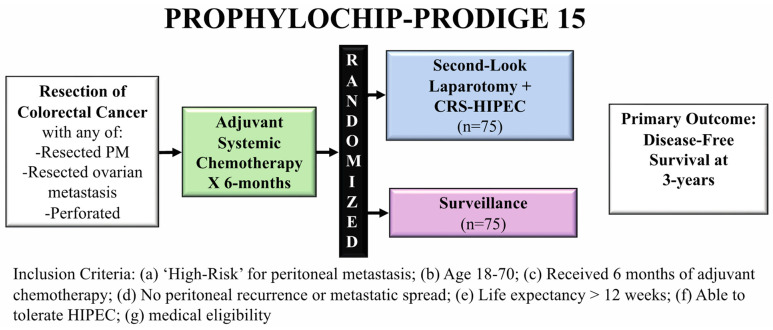
PROPHYLOCHIP-PRODIGE 15 trial schema comparing second-look laparotomy with CRS-HIPEC to surveillance in high-risk patients for peritoneal metastases.

The second trial, PROPHYLOCHIP-PRODIGE 15, examined the concept of second-look surgery and HIPEC in patients at high-risk of peritoneal recurrence, defined as having a synchronous PM that was resected, resected ovarian metastases, or perforated tumors [25]. In the trial, 150 patients underwent resection of the limited peritoneal disease and then received 6 months of systemic chemotherapy. Patients then underwent either second-look surgery or surveillance (Figure 4).

Second-look surgery consisted of performing an exploratory laparotomy, with CRS if PM was present, and HIPEC with intraperitoneal oxaliplatin for 30 min. They also received intravenous fluorouracil and leucovorin immediately before starting the HIPEC portion of the case (bidirectional therapy). The primary outcome was 3-year disease-free survival, and the authors found no difference between the experimental and surveillance arms (44% vs. 53%, respectively; hazard ratio 0.97, 95% CI: 0.61–1.56, *p* = 0.82). There was no difference in 5-year overall survival (second-look surgery 68% (55–79%) vs. surveillance 72% (60–82%)).

Although no post-operative mortality was reported in the second-look surgery arm, the rate of grade 3–4 adverse events was high at 40%. The authors concluded that second-look surgery did not improve 3-year disease-free survival but acknowledged there were potential confounders. The study had a lower recurrence rate than expected, potentially because over one-third of patients had perforated tumors, without peritoneal metastases, which would be considered “medium risk”. Despite the requirement that all included patients exhibit no signs of peritoneal disease on CT scan 6 months following the completion of chemotherapy, 52% of patients who underwent second-look laparotomy had peritoneal disease described by the operating surgeon (70% of which were confirmed pathologically). These results underline the difficulty of pre-operatively staging the peritoneum, especially for low volume disease, which could have influenced the results of this trial [26,27,28]. Again, the choice of oxaliplatin as the intraperitoneal perfusate may have obscured the benefit of this strategy for the same reasons noted for the PRODIGE 7 trial. Additionally, the authors hypothesized that while the intervention (HIPEC) in COLOPEC may have been too early, their protocol may have been too late, maintaining that there may be a potential benefit to “interval” second-look surgery.

The authors concluded that systematic second-look surgery did not improve disease-free survival compared to surveillance.

### 3.3. Conclusion to Early Interventions in High-Risk Patients for Peritoneal Metastases

Although COLOPEC and PROPHYLOCHIP were both negative studies in preventing peritoneal spread/recurrence, they offer a framework for additional hypothesis testing to mitigate the morbidity and mortality of CRC PM.

## 4. Future Directions

A number of clinical trials are in process to evaluate unanswered questions in the management of CRC PM. One trial of note, CAIRO6, seeks to evaluate perioperative systemic chemotherapy and CRS-HIPEC compared to CRS-HIPEC alone in patients with upfront resectable PM. In the Phase II portion of the study, the perioperative chemotherapy regimen appeared safe, although there were similar proportions of macroscopically complete CRS between study arms, perhaps suggesting a lack of benefit of preoperative therapy [29]. The results of the Phase III portion are eagerly awaited [30]. In addition, the GECOP-MMC trial is a phase IV randomized trial evaluating CRS alone compared to CRS-HIPEC with 90 min of MMC [31]. Importantly, this trial will be limited to patients with a PCI ≤ 20 who undergo complete cytoreduction, and the primary outcome is 3-year peritoneal recurrence-free survival. A similar study was also discussed at the Advanced Cancer Therapies 2022 Annual Meeting by the authors of PRODIGE 7. Together these trials promise to advance our knowledge on the optimal role and sequence for both systemic therapy and CRS-HIPEC in patients with CRC PM.

What appears clear is that a more complete understanding of tumor biology is needed to better comprehend this conflicting data. Beyond pathological determinants of poor tumor biology, novel biomarkers, such as plasma circulating tumor DNA (ctDNA), promise to improve our detection of, and treatment for, CRC PM [32]. Pre-operative ctDNA offers a potential avenue to improve selection, as detection of ctDNA has been associated with reduced disease-free survival, potentially indicating undiagnosed systemic disease or an increased potential for metastatic spread [33]. Furthermore, post-operative ctDNA has also been associated with decreased progression-free survival [34]. Improved platforms such as these will inform future clinical trials, helping to select the most efficacious regimens to individualize cancer care for this complex patient population.

## 5. Conclusions

Colorectal PM is a clinical problem commonly encountered in practice, which requires evaluation and management at specialized centers by experienced clinicians. Attention to proper patient selection, in line with recommendations established in the Chicago consensus, is paramount to success [35]. The studies described in this review show that adjuvant HIPEC and delayed second-look surgery using 30 min of intraperitoneal oxaliplatin are ineffective strategies to reduce PM/recurrence and improve survival in patients with CRC PM. Although early data supported CRS-HIPEC, results from the recent PRODIGE 7 study question the added benefit of HIPEC for this treatment strategy. These studies confirm the benefit of CRS, which should be considered the standard of care. Although many methodological critiques of PRODIGE 7 exist, the onus now appears to be on HIPEC enthusiasts to develop future trials required to resolve criticisms of the study and demonstrate any potential survival benefit of HIPEC for CRC PM.

## Abbreviations

Colorectal cancer (CRC); peritoneal metastases (PM); cytoreductive surgery (CRS); hyperthermic intraperitoneal chemotherapy (HIPEC); completeness of cytoreduction (CCR); mitomycin C (MMC); randomized control trial (RCT); 5-fluorouracil (5-FU); overall survival (OS); peritoneal carcinomatosis index (PCI); circulating tumor DNA (ctDNA).
